# CO_2_-breathing and piercing polymersomes as tunable and reversible nanocarriers

**DOI:** 10.1038/srep23624

**Published:** 2016-03-29

**Authors:** Anchao Feng, Jiamei Liang, Jinzhao Ji, Jinbo Dou, Shanfeng Wang, Jinying Yuan

**Affiliations:** 1 Key Lab of Organic Optoelectronics & Engineering, Department of Chemistry, Tsinghua University, Beijing 100084 (P.R. China); 2 Department of Materials Science and Engineering, The University of Tennessee, Knoxville, Tennessee 37996, United States

## Abstract

Despite numerous studies on utilizing polymeric vesicles as nanocapsules, fabrication of tunable molecular pathways on transportable vesicle walls remains challenging. Traditional methods for building penetrated channels on vesicular membrane surface often involve regulating the solvent polarity or photo-cross-linking. Herein, we developed a neat, green approach of stimulation by using CO_2_ gas as “molecular drill” to pierce macroporous structures on the membrane of polymersomes. By simply introducing CO_2_/N_2_ gases into the aqueous solution of self-assemblies without accumulating any byproducts, we observed two processes of polymeric shape transformation: “gas breathing” and “gas piercing.” Moreover, the pathways in terms of dimension and time were found to be adjustable simply by controlling the CO_2_ stimulation level for different functional encapsulated molecules in accumulation, transport, and releasing. CO_2_-breathing and piercing of polymersomes offers a promising functionality to tune nanocapsules for encapsulating and releasing fluorescent dyes and bioactive molecules in living systems and also a unique platform to mimic the structural formation of nucleus pore complex and the breathing process in human beings and animals.

Vesicles assembled by amphiphilic copolymers can act as microscopic sacs with molecular membrane structures and have been widely investigated in living and artificial systems^1^,^2^,^3^,^4^,^5^,^6^,^7^. Because of numerous possibilities for tailoring physicochemical and biological properties, some vesicular systems are desirable capsules for encapsulation and targeted delivery of drugs, fluorescent dyes, and biomolecules^8^,^9^. For these applications, it is critical to regulate reagent transport across the polymersome walls. Environmental stimuli are able to alter the permeability, integrity, and conformation of the polymersome walls^10^,^11^,^12^,^13^. It is also necessary to prepare polymer capsules with controllable membrane properties and capacity of transport. In nature, nucleus pore complex scattered over the nuclear envelope, as the gateway to connect cell nucleus and cytoplasm in eukaryotes, can control the diffusion of small molecules and ions and mediate the transport of proteins and RNAs^14^,^15^. To mimic nucleus pore complex, construction of porous molecularly self-assembled vesicles is fascinating and can regulate transport and encapsulate guest molecules at mild conditions. These porous vesicles are often formed by modulating the solvent polarity or using photo-cross-linking^16^,^17^,^18^,^19^, but no report is known to regard their morphological transformation. In order to mimic the real environment in a living body, it is crucial to study aqueous systems and exploit new trigger modes that can be applied to physiological conditions.

Carbon dioxide (CO_2_), a pivotal endogenous metabolite, is an ideal option for tuning the bilayer structure and studying the mechanism of the morphological transformation because it has good biocompatibility and membrane permeability. CO_2_ has thus been used as a stimulus to subtly adjust polymer vesicular size, permeability, stability, and morphologies, for example, vesicle swelling, transformation to micelles with inter-outer exchange, and formation of large-compound sacs^12^,^20^,^21^,^22^,^23^,^24^,^25^,^26^. Nevertheless, more complicated morphologies are needed to mimic the complex changes in living systems. To achieve this goal, advanced polymer structures are needed. For example, dendritic polymers with a highly branched three-dimensional architecture, including dendrimers and hyperbranched polymers, demonstrated their promise in preparing vesicles^27^,^28^. The resulted hyperbranched polymer vesicles were termed as branched-polymersomes (BPs)^29^ and have unique properties such as good stability, low permeability, giant and easily tuned vesicle size, appealing solution behaviours, and facile tailorability of the properties. Extensive studies are still needed for elucidating the mechanism for the formation of BPs and verifying the results obtained using dissipative particle dynamics (DPD) simulations^30^.

Here in this study, we designed and synthesized an amphiphilic dendritic star-block terpolymer with the ‘graft onto’ strategy using the combination of ring-opening polymerization (ROP), atom transfer radical polymerization (ATRP), and click chemistry. Started from a dendritic polyester, this terpolymer subsequently consisted of a hydrophobic poly(ε-caprolactone) (PCL) block, an intermediate CO_2_-sensitive bridging poly(*N*, *N*-diethylaminoethyl methacrylate) (PDEAEMA) block, and a hydrophilic poly(ethylene glycol) (PEG) end block, with an abbreviation of 220P-star-PCL-*b*-PDEAEMA-*b*-PEG. The biodegradable, semi-crystalline PCL with a low glass transition temperature (T_g_) of ~−60 °C was selected to explore new morphologies. This amphiphilic terpolymer can self-assemble into vesicular nanostructures in aqueous solutions with unique properties. When CO_2_ was introduced into the terpolymer aqueous solution, the PDEAEMA block was gradually protonated, leading to successive variation of the amphiphilic balance and continuous self-expansion of the polymersomes. More importantly, with stepwise tuning of the CO_2_ level, the membrane structure and permeability were modulated and macroporous vesicles were observed. Thus we speculated that CO_2_ not only can work as molecular driver for the “breathing” features of polymersome^20^,^22^, but also can perform like a “molecular drill” to manipulate valves in the vesicle membrane (Fig. 1). To the best knowledge of ours, it is the first example of constructing macroporous vesicles through “green” stimulus CO_2_ gas in a purely aqueous solution. Besides serving as nanocarriers in living systems, the vesicles with the ability of shape evolution are in many ways reminiscent of the structure of nucleus pore complex.

## Results and Discussion

### Preparation of self-assemblies in aqueous solution

**Figure 1:**
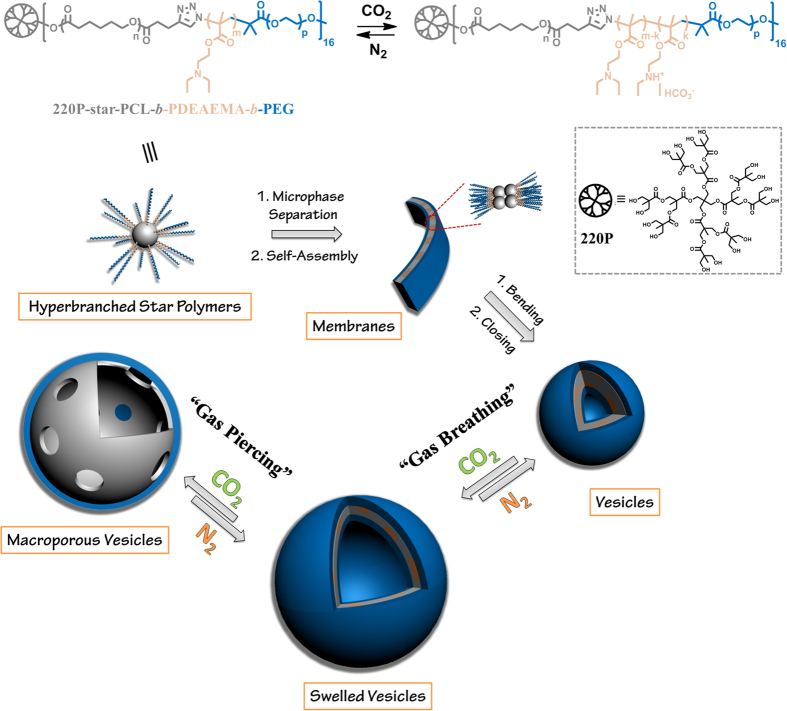
Schematic illustration of CO_2_-responsive vesicles with shape evolution. Gas-sensitive structural transition of amphiphilic dendritic star-block terpolymers (top). Terpolymer self-assembly into vesicles, reversible gas-driven controlled self-assembly and shape transformation (bottom).

**Figure 2:**
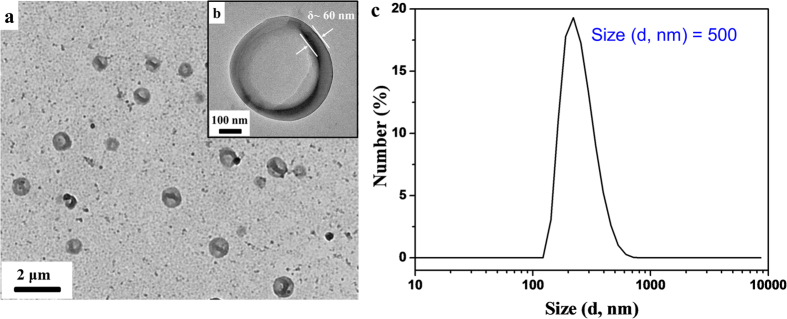
Self-assemblies of the terpolymer in its aqueous solution. (**a,b**) TEM images of the self-assemblies at different magnifications and (**c**) DLS curve to show the size distribution of the self-assemblies.

The 220P-star-PCL-*b*-PDEAEMA-*b*-PEG terpolymer had a number-average molecular weight (*M*_n_) of 61000 g/mol and a polydispersity index of 1.34. We used the nanoprecipitation method to prepare the polymer aqueous solution: 30 mg of the terpolymer were first dissolved in 1 mL THF, a good solvent to all the blocks; 9 mL deionized water was then injected at a slow rate of 0.9 mL/h to yield a translucent colloidal solution, and the organic phase was finally removed by dialysis. The strong Tyndall effect implied the formation of assemblies. Using the fluorescent probe method, the critical aggregation concentration (CAC) was determined as ~0.022 mg/mL (Supporting Information, Figure S7). Then transmission electron microscopy (TEM) was used to visualize the size and morphology of the assemblies, as shown in Fig. 2a,b. To enhance the contrast, polymer vesicles were stained with phosphotungstic acid (PTA) hydrate. The clear contrast between the dark periphery and the hollow centre indicated that these spheres were vesicular with a wall thickness of 54 nm and an average diameter of 472 nm. This result was consistent with the dynamic light scattering (DLS) result in Fig. 2c, which showed a peak at ~500 nm. The vesicles formed as the hydrophobic PCL and PDEAEMA segments mainly constituted the vesicle membrane, whereas the PEG segments were mainly the inner and outer hydrophilic parts, as illustrated in the vesicle structure in Fig. 1. The self-assembly mechanism of this terpolymer involves microphase separation, aggregation to bilayer membranes, and bending/closing to spherical vesicles. Such process had been well studied using DPD simulation and verified by many experimental results^31^,^32^.

### Gas-induced morphological transition

**Figure 3:**
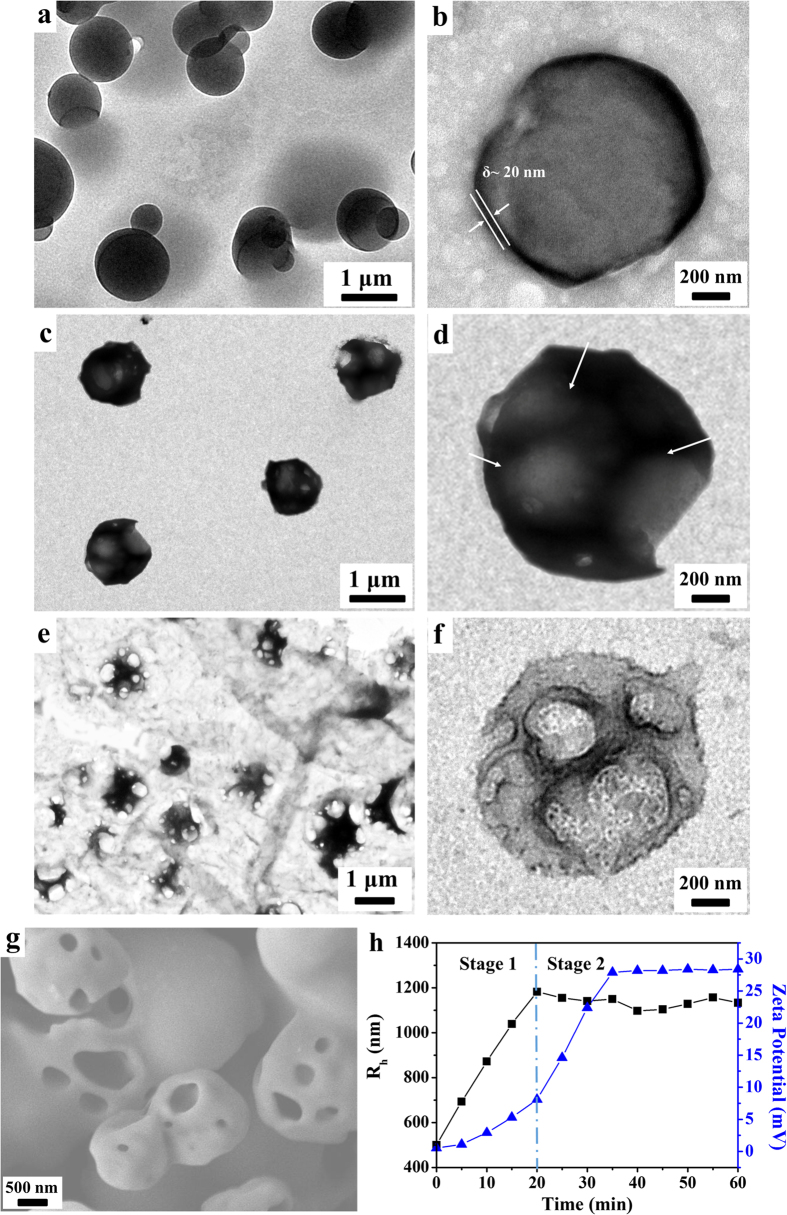
Self-assemblies of the terpolymer in its aqueous solution under CO_2_ stimulation. TEM images of the self-assemblies after CO_2_ treatment for (**a,b**) 20 min, (**c,d**) 30 min, and (**e,f**) 50 min at different magnifications and SEM image (**g**) after CO_2_ treatment for 50 min. (**h**) Rh and zeta potential as a function of CO_2_ aeration time in the period of 0–60 min.

We further explored whether the self-assemblies can undergo morphological changes upon exposure to CO_2_ by doing a time-lapsed measurement on the vesicles at different CO_2_ concentrations. A gas microflow pump was used to stabilize the aeration rate at ~20 mL/min and samples were collected at a fixed time interval of 5 min to observe the entire process. These vesicles started to expand upon CO_2_ treatment, as larger intact vesicles with a diameter of 1000 nm were observed after 20 min (Fig. 3a,b). Compared with the counterparts without CO_2_ stimulus, the vesicle volume increased strikingly by 700% and the wall thickness decreased to ~20 nm. In agreement with the TEM result, the vesicle size determined by DLS analysis, i.e., hydrodynamic radius R_h_, increased linearly with the gas stimulation time in the period of 0–20 min and reached a maximum of 1180 nm (Fig. 3h, stage 1). After the gas stimulation for 20 min, the diameter of the polymersomes did not change significantly and slight phase segregation appeared within the vesicle membrane, indicated by the clear contrast between the dark outline of the vesicle membrane and the grey internal patches pointed by white arrows in Fig. 3c,d. When the CO_2_ aeration time was prolonged to 50 min, macroporous vesicular structures were evident (Fig. 3e,f). More CO_2_ aeration before reaching saturation did not change the self-assembly morphology and such macroporous vesicles were stable for at least one week. Correspondingly, R_h_ remained about the same in the period of 20–60 min (Fig. 3h, stage 2). The surface morphology of these self-assemblies was further examined using scanning electron microscopy (SEM) to confirm that the low-contrast patches on the membrane surface were indeed macropores, as demonstrated in Fig. 3g. In the case of 220P-star-PCL-*b*-PDEAEMA-*b*-PEG terpolymer, this series of morphology changes must be associated with protonation of PDEAEMA in CO_2_ environment. To validate this viewpoint, the change of surface charge was further detected by a zeta potentiometer. The initial vesicles presented a weak surface charge of +0.5 mV. Then the zeta potential climbed steadily to +8.1 mV upon CO_2_ treatment for 20 min. When the CO_2_ addition was prolonged to 35 min, an immediate increase in the surface charge to +27.9 mV was observed, indicating that massive protonated PDEAEMA chains were exposed on the surface of vesicles. Thereafter, the surface charge remained around +28.2 mV even when the CO_2_ addition was further prolonged to 60 min (Fig. 3h). Together with the results of TEM, SEM, and DLS, it can be concluded that the protonated PDEAEMA chains were distributed on the surface of the generated macropores and ensured a stable topological structure of the macroporous vesicles.

**Figure 4:**
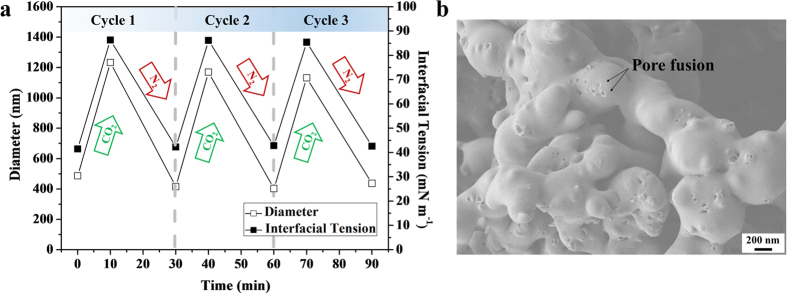
Gas-controlled reversible morphological transition. (**a**) Variation in the diameter and interfacial tension of the terpolymer self-assemblies upon alternating CO_2_/N_2_ stimulation. (**b**) SEM images of the self-assemblies after N_2_ treatment.

The terpolymer self-assemblies with a relatively compact structure displayed good stability and reversibility in CO_2_/N_2_ cyclic tests. As demonstrated in Fig. 4a, alternating changes in the vesicle diameter and interfacial tension in 10 mL self-assembly solution (1 mg/mL) upon CO_2_/N_2_ treatment confirmed their reversible swelling and shrinkage for at least three loops. When CO_2_ was injected to the system for 10 min, an increase in interfacial tension accompanied with the size increment indicated expansion of the terpolymer vesicles. Oppositely, the interfacial tension and vesicle size decreased back to the original levels when N_2_ gas was introduced to expel CO_2_ from the system at the same aeration rate for 20 min, which consequently induced vesicle shrinkage and closure or fusion of the macropores in the vesicle wall. This mechanism of this reversible morphological transformation was further confirmed by the SEM image of the vesciles after N_2_ treatment in Fig. 4b. The above results indicated that multiple morphological transitions can be realized in a controllable and reversible manner by simply tuning the CO_2_ level, in which CO_2_ acted as a molecular “driver” and “drill” to manipulate the molecular pathways of evolution.

**Figure 5:**
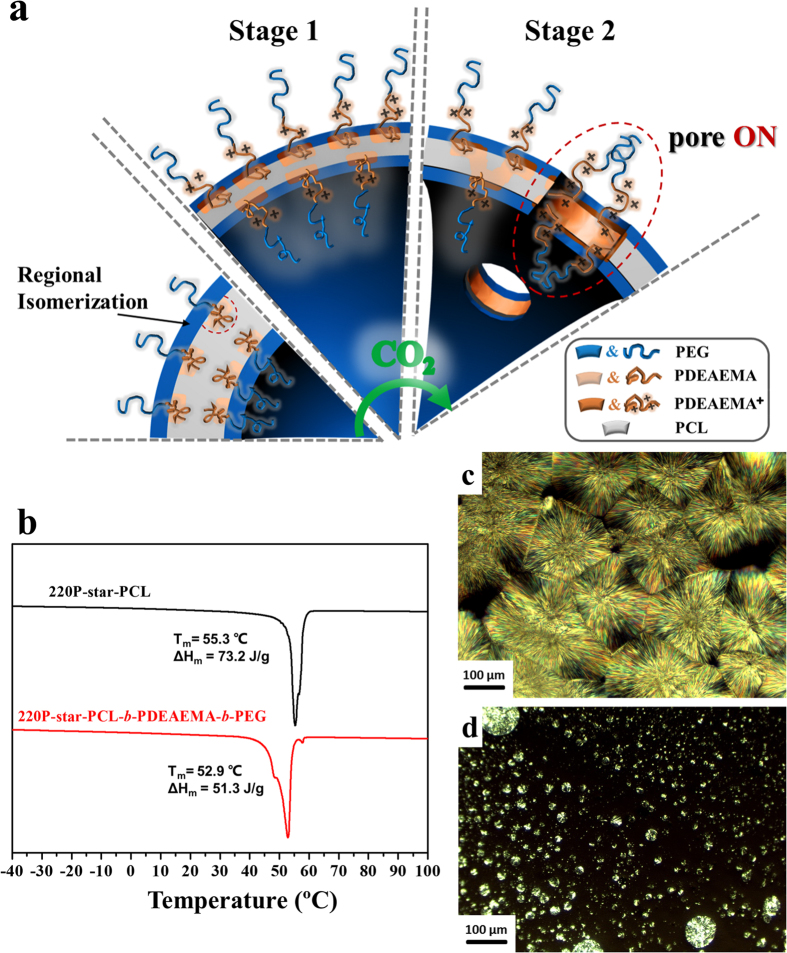
Regional isomerization in the terpolymer. (**a**) Schematic illustrations of the changes of the vesicular radius and internal regional isomerization on vesicle membrane under CO_2_ stimulation. (**b**) DSC curves of 220P-star-PCL and 220P-star-PCL-*b*-PDEAEMA-*b*-PEG. (**c,d**) POM images of 220P-star-PCL (**c**) and 220P-star-PCL-*b*-PDEAEMA-*b*-PEG (**d**) crystallized at 45 °C.

The mechanism on how CO_2_ drove the shape evolution of the terpolymer self-assemblies is proposed as follows. First, the PDEAEMA block was hydrophobic to some extent and it together with PCL formed the membrane of the self-assembled vesicles. As for the asymmetrical copolymers (i.e. PDEAEMA and PCL with different volume fractions), the insoluble domains are present in internal morphology^33^,^34^. During the formation of the membrane, regional isomerization occurred as the result of different thermodynamic properties between PDEAEMA and PCL segments, as depicted in Fig. 5a. The thermal properties and crystallographic features of 220P-star-PCL-*b*-PDEAEMA-*b*-PEG were examined to understand this regional isomerization. Differential Scanning Calorimetrical (DSC) curves in the heating run (Fig. 5b) showed that the melting temperature (T_m_) and the heat of fusion (ΔH_m_) of the terpolymer were both lower than those of 220P-star-PCL, as can be attributed to crystalline imperfection^35^. In dendritic terpolymers, the motions of the PCL segments were hindered by the steric interference. PDEAEMA, an amorphous polymer^36^, has poor miscibility with crystalline PCL. Thus the PDEAEMA blocks tended to be phase-separated from the PCL crystalline regions, resulting in a PCL-rich phase and a PDEAEMA-rich one^37^,^38^. In order to support the thermal analysis using DSC, isothermal crystallization of 220P-star-PCL-*b*-PDEAEMA-*b*-PEG and 220P-star-PCL were further conducted and the crystalline morphologies were observed under a Polarized Optical Microscope (POM). Typical PCL spherulites were seen in the POM image of 220P-star-PCL in Fig. 5c. In clear contrast, no evident PCL crystalline structures were found in 220P-star-PCL-*b*-PDEAEMA-*b*-PEG in Fig. 5d, whereas typical PEG spherulites^39^,^40^,^41^ appeared instead. The above results suggested that PCL crystallization in the terpolymer was strongly inhibited by the amorphous PDEAEMA block while the PEG block in the chain end was still able to crystallize.

When CO_2_ was introduced into the solution, part of the PDEAEMA blocks gradually transformed from a neutral, collapsed state to a protonated, stretched, and hydrophilic state (Fig. 5a, stage 1). The polymersomes were obliged to expand gradually, accompanied by a decrease in the membrane thickness to attain a lower interfacial free energy. With increasing the concentration of CO_2_, the repulsion among the positive charges and hydrophilicity of protonated PDEAEMA segments resulted in the formation of pores in the membrane surface (Fig. 5a, stage 2). Meanwhile, the PCL-rich matrix contributed to the significant stability as the skeleton. In the presence of N_2_, the protonated PDEAEMA segments were deprotonated and these vesicles then shrank back to their initial size.

### Controlled release from the macroporous vesicles

**Figure 6:**
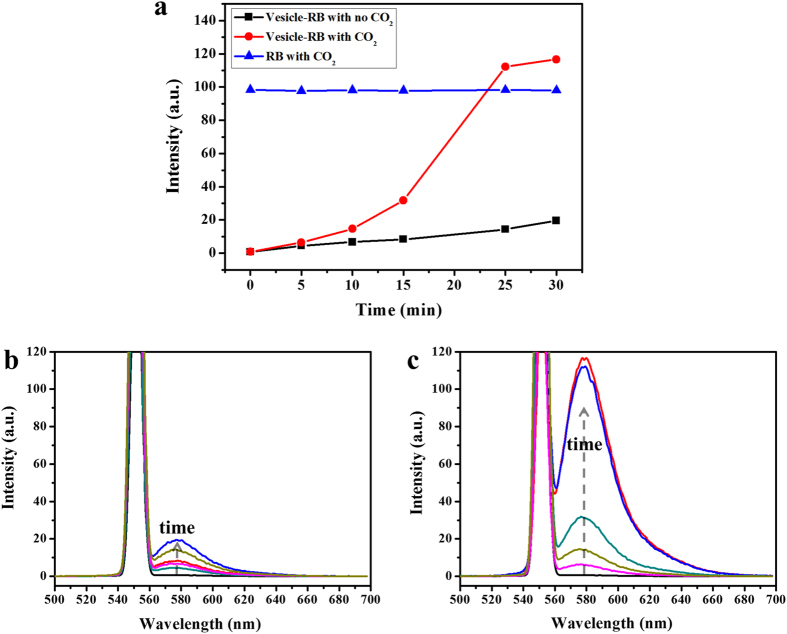
Controlled release of RB from the vesicles. (**a**) Fluorescence intensity at the wavelength of 580 nm as a function of time for vesicles containing RB with (red circles) or without (black squares) CO_2_ stimulation and RB aqueous solutionin the prensence of CO_2_ stimulation. Fluorescence emission spectra of (**b**) vesicle + RB without CO_2_ stimulus and (**c**) vesicle + RB with CO_2_ stimulation.

The penetrable and robust nanovesicles of the terpolymer suggested as excellent vehicles for encapsulation and carrying of fluorescent dyes and bioactive molecules into living systems. Fluorescent Rhodamine B (RB) as a model dye has been encapsulated into the interior of vesicles^42^ with only weak interaction with polymer matrix. RB-loaded BPs were prepared as the solution of the self-assemblies was dialyzed against the deionized water until the water outside the dialysis tube exhibited negligible RB fluorescence. The concentration of RB in the solution was controlled under 10^−3^ M to be positively correlated with the fluorescence intensity. First, the aqueous solution of RB was treated with CO_2_ for 30 min to confirm that the acidic gas had no obvious effect on the fluorescent property of RB (Fig. 6a, blue line). Then to understand the release behaviour of RB, the fluorescence of the solution was examined when bubbling with CO_2_, compared with the control group without CO_2_ stimulus. It can be seen from the fluorescence spectra in Fig. 6b,c that the intensity at the highest emission wavelength of ~580 nm increased gradually with time for both groups. To reveal the variation tendency more visually, the fluorescence intensities at the highest emission wavelength of 580 nm were presented as functions of time in Fig. 6a. When the CO_2_ stimulus was within 15 min, the release from the nanocapsules was low because of their compact membrane structure. Then an abrupt release occurred at ~15 min, and the released fraction of RB reached ~100% in a short period of 10 min. As discussed earlier, pores inevitably opened and built channels for rapid release of guest molecules at this critical concentration of CO_2_. The coincidence between the morphological evolution and release profile indicated that the RB release time could be precisely tuned through varying the CO_2_ concentration and flow rate. Because of the high release rate, the amount of encapsulated guest molecules in these nanocarriers does not need to be more than required, which is especially useful for costly or lethal ones.

The cytocompatibility of the terpolymer and its two precursors, 220P-star-PCL and PEG-*b*-PDEAEMA-N_3_, was also evaluated using primary rat aortic smooth muscle cells (SMCs) and ~100% cell viability at days 1 and 4 were found for all these three samples when compared with the cell culture well, the positive control (Supporting Information, Figure S8). Thus, we can draw conclusion that these BPs can serve as nanocapsules for functional fluorescent dyes and bioactive molecules in living systems over tunable time and quantity through CO_2_ stimulus without releasing or generating cytotoxic components. Further *in vivo* biocompatibility and functionalities of the terpolymer and its self-assembled vesicles will be examined in animal models.

## Discussion

In summary, we have developed a CO_2_-sensitive amphiphilic terpolymer 220P-star-PCL-*b*-PDEAEMA-*b*-PEG that can spontaneously form vesicles in aqueous media. CO_2_ can tune the size of these vesicles and pierce macropores on the wall by controlling the degree of protonation of the PDEAEMA block. Without any organic solvent and reaction by-products, we for the first time observed self-assembled macroporous vesicles using a green procedure that only involves introduction and release of inactive gases such as CO_2_ and N_2_. The *in situ* prepared pores on the vesicular membrane offer controllable pathways to encapsulated molecules for accumulation, transport, and releasing, simply through modulating the CO_2_ concentration. Such shape evolution of the vesicles supplies a platform to investigate the mechanism of the structural formation of nucleus pore complex and to mimic the breathing process in human beings and animals. These CO_2_-breathing and piercing polymersomes also function as nanocapsules for fluorescent dyes and bioactive molecules in living systems.

## Methods

### Materials

Hyperbranched polyester initiator, 220P, with an *M*_n_ of ca. 1600 g/mol (Weihai CY Dendrimer Technology Co., Ltd), mono-methoxyl PEG with an *M*_n_ of ca. 2000 g/mol (Aldrich), ε-caprolactone (CL, Acros, 99%), stannous octoate (Sn(Oct)_2_, Aladdin, 95%), 4-pentynoic acid (J&K Scientific Ltd., 95%), *N*, *N*-dicyclohexylcarbodiimide (DCC, J&K Scientific Ltd., 99%), 4-dimethylaminopyridine (DMAP, TCI, 99%), 2-bromoisobutyryl bromide (Acros, 98%), trimethylamine (TCI, Shanghai, 99%), tris(2-dimethylaminoethyl)amine (ME6-TRAN, Alfa Aesar, 99%), *N*,*N*,*N*′,*N*″,*N*″-pentamethyldiethylenetriamine (PMDETA, Aldrich, 99%), dichloromethane (CH_2_Cl_2_, J&K Scientific Ltd., 99.8%), Ethylenediaminetetraacetic acid disodium salt (EDTA-2Na, J&K Scientific Ltd.) and *N*,*N*-dimethylformamide (DMF, J&K Scientific Ltd., 99.9%) were used as received. 2-diethylaminoethyl methacrylate (DEAEMA, J&K Scientific Ltd., 99.5%) was purified by passing through a basic alumina column. Copper (I) bromide (CuBr, J&K Scientific Ltd., 98%) was purified by stirring with acetic acid overnight, filtered, and then successively washed with acetone and ethanol until it turned white, followed by complete drying in a vacuum oven at room temperature. Other solvents were purchased from Beijing Chemical Reagent Co. Ltd (China) and used as received. Deionized and distilled water was utilized throughout the study.

### Measurements

^1^H Nuclear Magnetic Resonance (NMR) spectra of the polymers were obtained from a JEOL JNM-ECA400 (400 MHz) and a JEOL JNM-ECA600 (600 MHz) spectrometer. The Fourier-transform Infrared (FTIR) absorption spectra of the polymers were recorded at 30 scans with a spectral resolution of 1 cm^−1^ on an AVATAR 360 ESP FTIR spectrometer. Gel-permeation chromatographic (GPC) analyses of the polymers were performed using DMF as the eluent. The GPC system was composed of a Shimadzu LC-20AD pump system, a MZ-Gel SDplus 10.0 μm guard column (50 mm × 8.0 mm, 102 Å) followed by a MZ -Gel SDplus 5.0 μm bead-size column (50–106 Å, linear), and a Shimadzu RID-10A refractive index detector. The GPC system was calibrated with monodisperse polystyrene standards with M_w_ of 200 to 10^6^ g/mol. The average radius and size distribution of the self-assemblies in aqueous solutions were analyzed using a Malvern 3000HS Zetasizer with a monochromatic coherent He–Ne laser (633 nm) as the light source and a detector to detect scattered light at an angle of 90°. The zeta-potential of the fully dialyzed polymer aggregated solutions was detected using the same Malvern 3000HS Zetasizer and the potential was determined in KCl aqueous media (5.0 mM) at 25 °C with a given gas stimulus. TEM images of the self-assemblies were obtained from a JEM-2010 microscope with an accelerating voltage of 120 kV and an H-7650B microscope with an accelerating voltage of 80 kV. The samples were prepared by drop-coating the aqueous solution on a carbon-coated copper grid and staining with 0.1% PTA hydrate. Interfacial tension was measured using a high-sensitive microelectromechanical balance system (Dataphysics DCAT21, Germany). The length and width of the plate for detection were 19.9 and 0.2 mm, respectively. The motor speed to drive the plate was 1.00 mm/s. The fluorescence emission intensity of the polymer aqueous solution was measured on a Jasco F-6500 (Shimadzu, Japan) at the excitation wavelength of 550 nm in a thermostatically controlled cuvette. DSC measurements were carried out on a Perkin Elmer Diamond differential scanning calorimeter in a N_2_ atmosphere and the samples were first heated from room temperature to 120 °C and cooled to −80 °C at 10 °C/min. Then a subsequent heating run was performed from −80 to 120 °C at 10 °C/min. The polymer films for crystallization test were prepared as follows: about 200 μL of the 220P-star-PCL or 220P-star-PCL-*b*-PDEAEMA-*b*-PEG solution in CH_2_Cl_2_ (1 g/10 mL) was spin-coated onto a round glass coverslip (15 mm, diameter) at a spin rate of 1000 rpm for 1 min at room temperature using a single-wafer spin processor (Laurell Technologies, North Wales, PA). After the polymer films were fully dried in a vacuum oven, they were melted on a heating plate at 80 °C for 5 min and then quickly transferred to a hot stage at 45 °C for isothermal crystallization and visualization under a POM (Nikon Eclipse, E600, Japan) until the film was fully covered by spherulites or the spherulites did not grow for over 5 h.

### Synthesis of 220P-star-PCL and 220P-star-PCL-yne

220P-*star*-PCL was prepared via ROP of CL using 220P as the macroinitiator and Sn(Oct)_2_ as the catalyst. 220P (0.21 g, 2.1 mmol of hydroxyl moietes) and CL (3.80 g, 33 mmol) were charged into a dry flask and stirred for 15 min. Sn(Oct)_2_ (0.20 g, 0.49 mmol) in anhydrous toluene was added into the flask, followed by three exhausting-refilling cycles. The reaction was carried out under stirring for 23 h at 120 °C. After being cooled to room temperature, the mixture was dissolved in ~10 mL of CH_2_Cl_2_ and precipitated into cold methanol. The product was dried in a vacuum oven at room temperature for 24 h, yielding 2.3 g (0.04 mmol, 57%) of white powder. *M*_n,NMR_ = 56.3 kg/mol, *M*_n,GPC_ = 38.1 kg/mol, *M*_w_/*M*_n_ = 1.45. ^1^H NMR (CDCl_3_, 400 MHz) (Figure S1a): *δ*_H_ (ppm) = 4.06 (-C***H***_***2***_-OOC-), 3.65 (-C***H***_***2***_-OH), 2.31 (-OOC-C***H***_***2***_-), 1.65 (-CH_2_-C***H***_***2***_-CH_2_-C***H***_***2***_-CH_2_-), 1.38 (-CH_2_-CH_2_-C***H***_***2***_-CH_2_-CH_2_-).

220P-*star*-PCL-yne was prepared as follows. 220P-*star*-PCL (1.44 g, 0.41 mmol of hydroxyl moieties), 4-pentynoic acid (0.69 g, 7 mmol), DMAP (185 mg, 1.5 mmol), 40 mL of CH_2_Cl_2_ and DCC (739 mg, 3.6 mmol) were added into a flask. The reaction was carried out at room temperature for 41 h. After the reaction, the mixture was diluted with CH_2_Cl_2_ and successively washed with saturated aqueous NaHCO_3_ and deionized water, three times each. The organic layer was collected, dried with anhydrous MgSO_4_ for 2 h and filtered, and then the filtrate was concentrated and precipitated in cold diethyl ether. The product was dried in a vacuum oven at room temperature for 14 h, yielding 0.6 g (0.01 mmol, 40%) of white powder. *M*_n,NMR_ = 57.4 kg/mol, *M*_n,GPC_ = 42.9 kg/mol, *M*_w_/*M*_n_ = 1.48. ^1^H NMR (CDCl_3_, 400 MHz) (Figure S1b): *δ*_H_ (ppm) = 4.06 (-C***H***_***2***_-OOC-), 2.53 (-C≡C***H***), 2.31 (-OOC-C***H***_***2***_-), 1.65 (-CH_2_-C***H***_***2***_-CH_2_-C***H***_***2***_-CH_2_-), 1.38 (-CH_2_-CH_2_-C***H***_***2***_-CH_2_-CH_2_-).

### Synthesis of PEG-Br macroinitiator and PEG-*b*-PDEAEMA-Br copolymer

PEG (12.01 g, 6.0 mmol) was dissolved in 80 mL of toluene, which was dried using azeotropic distillation. When the reaction system was cooled to room temperature, trimethylamine (0.60 g, 5.9 mmol) in 50 mL of CH_2_Cl_2_ was added into the solution. The reaction system was further cooled to 0 °C and then 2-bromoisobutyryl bromide (4.06 g, 17.6 mmol) in 13 mL of CH_2_Cl_2_ was added dropwise into the reaction solution in 1 h. After the reaction was stirred at room temperature for another 21 h, the mixture was washed three times with saturated aqueous NaHCO_3_ and another three times with deionized water. The organic layer was collected, dried with anhydrous MgSO_4_, concentrated, and precipitated in cold diethyl ether. The product was dried in a vacuum oven at room temperature for 12 h, yielding 10 g (4.8 mmol, 80%) of white powder. *M*_n,NMR_ = 2100 g/mol, *M*_n,GPC_ = 2800 g/mol, *M*_w_/*M*_n_ = 1.12. ^1^H NMR (CDCl_3_, 400 MHz) (Figure S2): *δ*_H_ (ppm) = 4.31 (-C***H***_***2***_-OOC-), 3.64 (-C***H***_***2***_-O-), 3.38 (-O-C***H***_***3***_), 1.94 (-C(C***H***_***3***_)_2_Br).

PEG-*b*-PDEAEMA-Br was synthesized by single electron transfer living radical polymerization (SET-LRP) with PEG-Br as the macroinitiator. DEAEMA (3.7 g, 20 mmol), PEG-Br (0.87 g, 0.4 mmol), 10 mL of anhydrous DMSO and Me_6_-TRAN (9.2 mg, 0.04 mmol) were introduced into a flask. After N_2_ was bubbled for 15 min, copper wire bent into a spiral was added into the mixture, followed by bubbling N_2_ for another 5 min. The reaction was carried out for 20 h at room temperature with N_2_ protection. After the mixture was dialyzed in methanol for 28 h, the product was evaporated and dried in a vacuum oven at room temperature for 24 h, yielding 1.7 g (0.23 mmol) of light green sticky solid. *M*_n,NMR_ = 7500 g/mol, *M*_n,GPC_ = 4000 g/mol, *M*_w_/*M*_n_ = 1.28. ^1^H NMR (CDCl_3_, 400 MHz) (Figure S3): *δ*_H_ (ppm) = 3.99 (-COO-C***H***_***2***_-), 3.64 (-C***H***_***2***_-O-), 3.38 (-O-C***H***_***3***_), 2.70 (-CH_2_-C***H***_***2***_-N-), 2.57 (-N-(C***H***_***2***_)_2_-), 1.80–1.94 (-C(CH_3_)-C***H***_***2***_-C(CH_3_)-Br), 1.04 (-N-(CH_2_-C***H***_***3***_)_2_), 0.90 (Br-C(CH_3_)-C***H***_***3***_).

### Synthesis of PEG-*b*-PDEAEMA-N_3_

PEG-*b*-PDEAEMA-N_3_ was prepared as follows: PEG-*b*-PDEAEMA-Br (1.00 g, 0.13 mmol), NaN_3_ (29 mg, 0.45 mmol), and 20 mL of DMF were charged in a round-bottom flask. The reaction was carried out at room temperature for 39 h. After the mixture was dialyzed in deionized water for 30 h, the product was freeze-dried, yielding 0.7 g (73%) of solid. FTIR (KBr, Figure S4): 2970, 2895, 2798 (ν_C–H_), 2112 (ν_azdio group_), 1730 (ν_C=O_).

### Synthesis of 220P-star-PCL-*b*-PDEAEMA-*b*-PEG

PEG-*b*-PDEAEMA-N_3_ (0.50 g, 66 μmol), PCL-yne (0.22 g, 55 μmol of alkynyl groups), 10 mL of anhydrous DMF, PMDETA (24 mg, 137.5 μmol), and CuBr (16 mg, 110 μmol) were introduced into a flask, followed by three exhausting-refilling cycles. The mixture reacted at 65 °C for 48 h. After being cooled to room temperature, the mixture was dialyzed in DMF for 24 h, and then dialyzed in EDTA-2Na aqueous solution until the color of the product turned white. Finally, the product was freeze-dried, yielding 0.62 g (33%). *M*_n,NMR_ = 120000 g/mol, *M*_n,GPC_ = 61000 g/mol, *M*_w_/*M*_n_ = 1.33. ^1^H NMR (CDCl_3_, 400 MHz) (Figure S5): *δ*_H_ (ppm) = 4.06 (-C***H***_***2***_-OOC-R), 3.99 (-COO-C***H***_***2***_-), 3.64 (-C***H***_***2***_-O-), 2.70 (-CH_2_-C***H***_***2***_-N-), 2.57 (-N-(C***H***_***2***_)_2_-), 2.31 (-OOC-C***H***_***2***_-), 1.80–1.94 (-C-C***H***_***2***_-C-), 1.65 (-CH_2_-C***H***_***2***_-CH_2_-C***H***_***2***_-CH_2_-), 1.38 (-CH_2_-CH_2_-C***H***_***2***_-CH_2_-CH_2_-), 1.04 (-N-(CH_2_-C***H***_***3***_)_2_), 0.90 (-C-C***H***_***3***_).

### Cytotoxicity

About 20 mg of polymer powder was sterilized in 70% ethanol solution for 5 h and completely dried in vacuum. The cytotoxicity evaluation was performed by culturing primary rat aortic SMCs at a density of ~15,000 cells/cm^2^ in a 24-well plate with 1 mL of primary medium, which consisted of high-glucose Dulbecco’s modified eagle medium (DMEM) with 10% fetal bovine serum, and 1% penicillin/streptomycin. The polymer samples, placed in the trans-wells, were immersed in the culture medium and exposed to SMCs for 1 and 4 days. Wells seeded with SMCs at the same density but without polymer samples were used as the positive control. The number of viable cells was obtained from a colorimetric cell metabolic assay based on the MTS tetrazolium compound and the UV absorbance at 490 nm (CellTiter 96 Aqueous One Solution, Promega, Madison, WI). Cell viability was then quantified by normalizing the average cell number in the sample wells by the value from the positive control wells.

## Additional Information

**How to cite this article**: Feng, A. *et al.* CO_2_-breathing and piercing polymersomes as tunable and reversible nanocarriers. *Sci. Rep.* **6**, 23624; doi: 10.1038/srep23624 (2016).

## Supplementary Material

Supplementary Information
