# Supplementary material for: CO2-breathing and piercing polymersomes as tunable and reversible nanocarriers
Source: Sci Rep. 2016 Mar 29;6:23624. doi: 10.1038/srep23624 (PMC4810324; doi:10.1038/srep23624)
Supplement: Supplementary Information [file srep23624-s1.pdf]

Supporting information

CO_2-breathing and piercing polymersomes as tunable and reversible nanocarriers

Anchao Feng[1], Jiamei Liang[1], Jinzhao Ji[1], Jinbo Dou[2], Shanfeng Wang[2] & Jinying Yuan[1]

1. Key Lab of Organic Optoelectronics & Engineering Department of Chemistry, Tsinghua University, Beijing 100084 (P.R. China).

2. Department of Materials Science and Engineering, The University of Tennessee, Knoxville, Tennessee 37996, United States.

Correspondence and requests for materials should be addressed to J. Y. (email: yuanjy@mail.tsinghua.edu.cn) or S. W. (email: swang16@utk.edu).

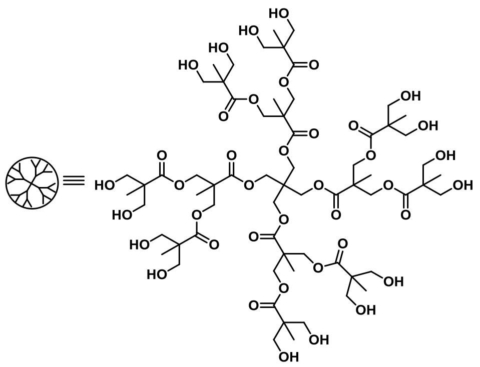

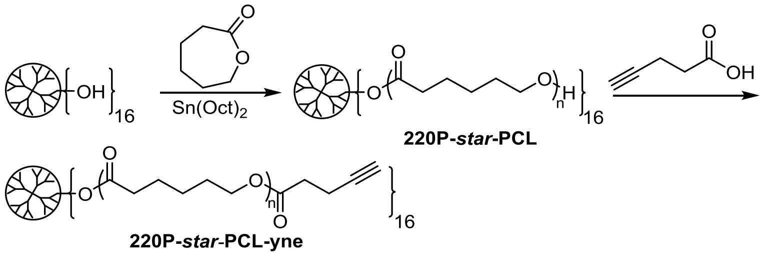

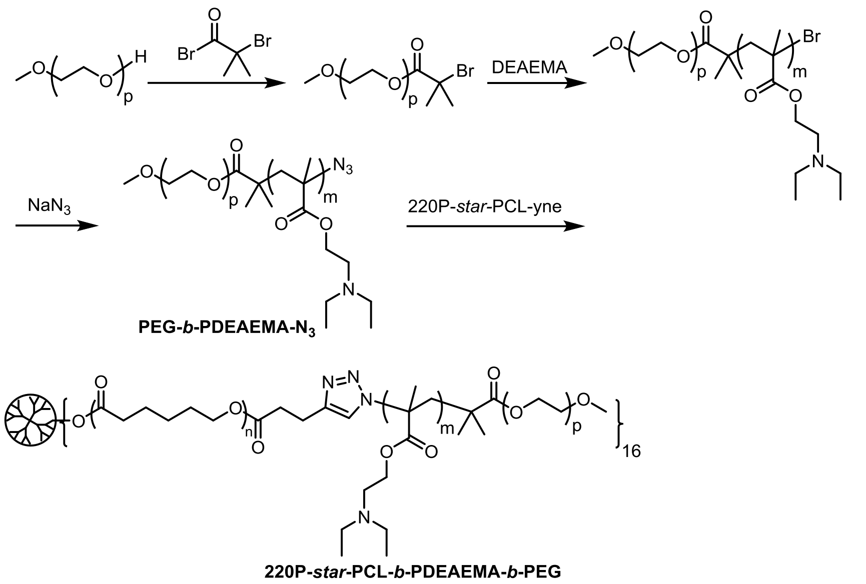

Figure S1. Synthetic route of the terpolymer 220P-star-PCL-*b*-PDEAEMA-*b*-PEG by the combination of ROP, ATRP, and click chemistry.

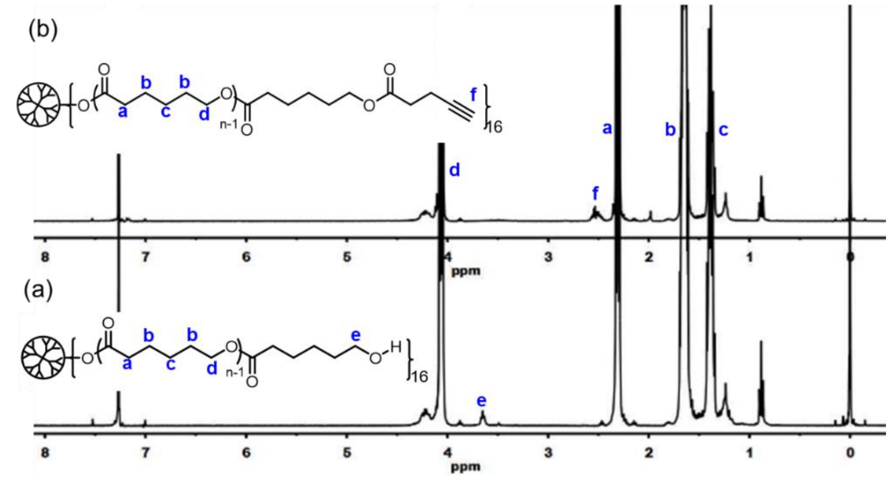

Figure S2. ^{1}H NMR spectrum of 220P-*star*-PCL and 220P-*star*-PCL-yne in $CDCl_3$.

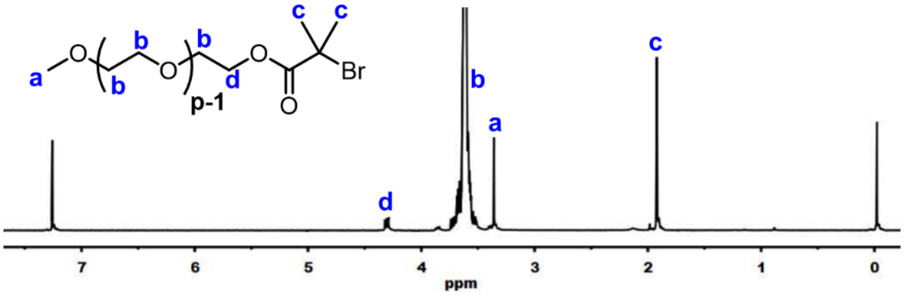

Figure S3. ^{1}H NMR spectrum of PEG-Br in $CDCl_3$.

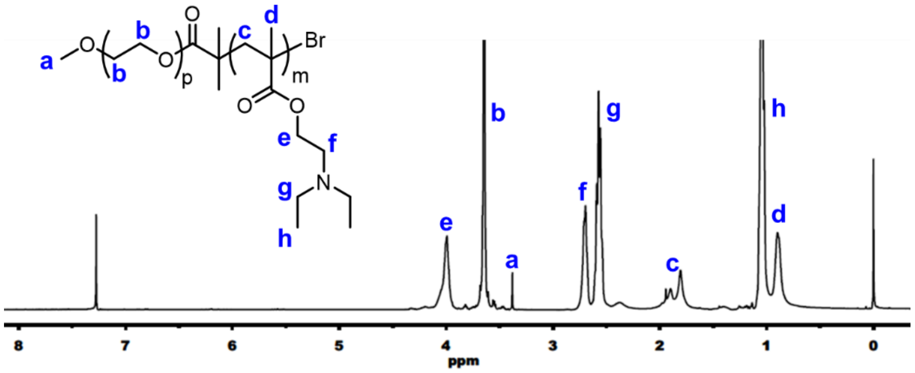

Figure S4. ^{1}H NMR spectrum of PEG-*b*-PDEAEMA-Br in $CDCl_3$.

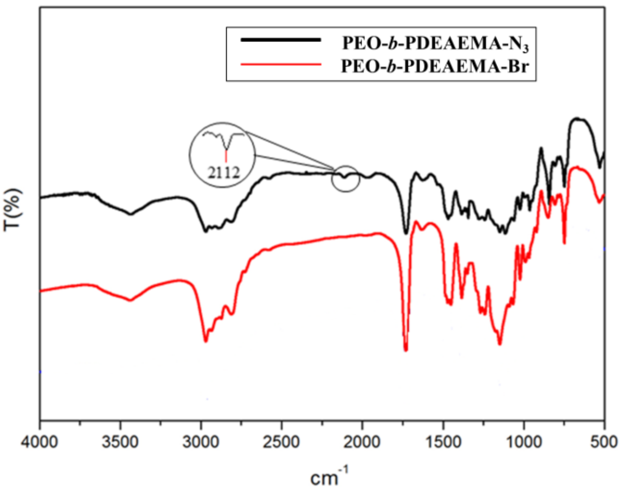

Figure S5. FT-IR spectrum of PEG-*b*-PDEAEMA-N_3 and PEG-*b*-PDEAEMA-Br.

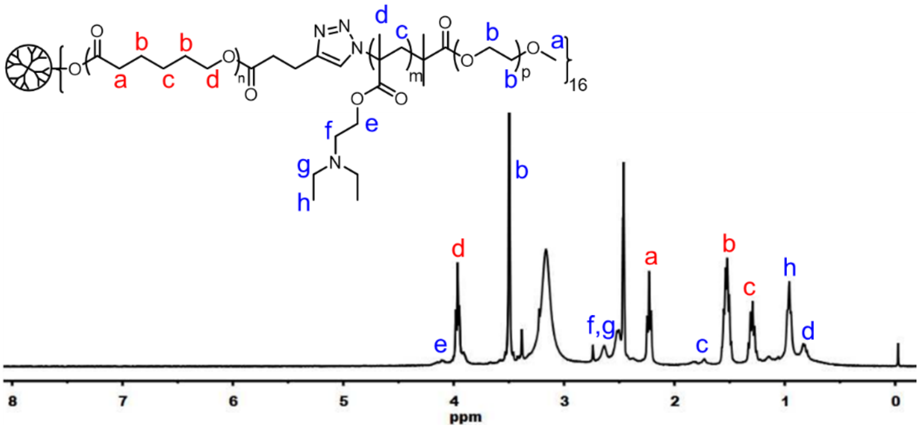

Figure S6. ^{1}H NMR spectrum of 220P-*star*-PCL-*b*-PDEAEMA-*b*-PEG in $CDCl_3$ at 80 ℃.

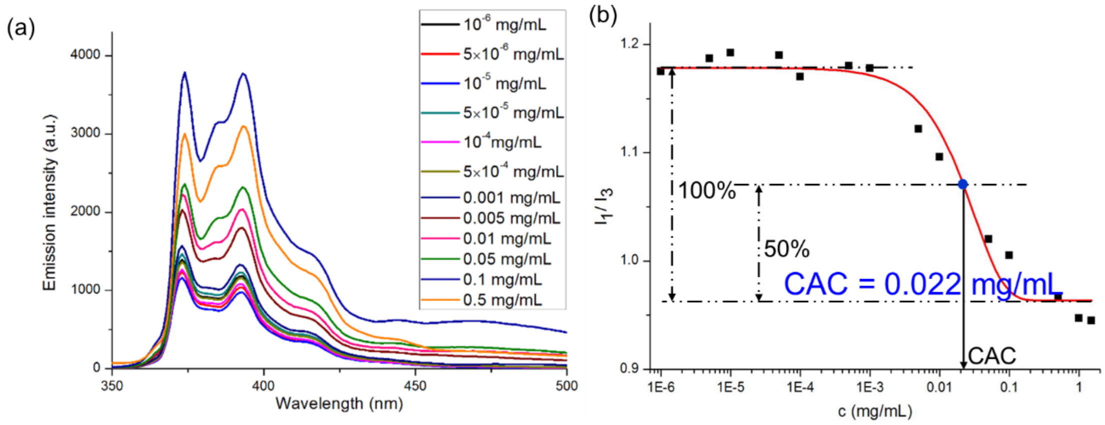

Figure S7. Determination of the critical aggregate concentration (CAC) for the terpolymer using the fluorescent method with pyrene as a probe[1]. Aqueous solutions of 220P-star-PCL-*b*-PDEAEMA-*b*-PEG self-assemblies at different concentrations were prepared, as well as a 5 mg/mL pyrene solution in acetone. To each of the aqueous solutions (2 mL), 20 μL stock solution of pyrene was added. The solutions were sonication for 5 min before fluorescent emission measurements. For each spectrum obtained, the intensity ratio of the first and third peaks, I_1/I_3 was calculated. The ratio data were well fitted by a Boltzmann-type sigmoid and the CAC was chosen as the concentration at which I_1/I_3 decreased to 50%. The results showed that the CAC was ~0.022 mg/mL.

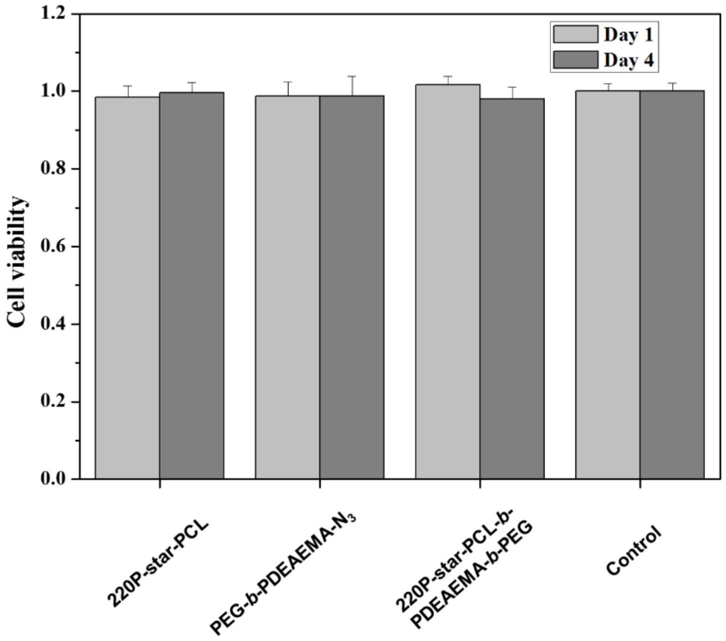

Figure S8. Cytotoxicity evaluation of the terpolymer and its two pre-cursors, 220P-star-PCL and PEG-*b*-PDEAEMA-N_3, with the positive

control as the reference for normalization.

Notes and references

1. Aguiar J, Carpena P, Molina-Bolívar JA, Carnero Ruiz C. On the determination of the critical micelle concentration by the pyrene 1:3 ratio method. *J. Colloid Interf. Sci.* **258**, 116-122 (2003).
